# Gallstone disease, diabetes, calcium, triglycerides, smoking and alcohol consumption and pancreatitis risk: Mendelian randomization study

**DOI:** 10.1038/s41525-021-00189-6

**Published:** 2021-03-29

**Authors:** Shuai Yuan, Edward L. Giovannucci, Susanna C. Larsson

**Affiliations:** 1 Unit of Cardiovascular and Nutritional Epidemiology, Institute of Environmental Medicine, Karolinska Institutet, Stockholm, Sweden; 2 Channing Division of Network Medicine, Department of Medicine, Brigham and Women’s Hospital and Harvard Medical School, Boston, MA USA; 3 Department of Epidemiology, Harvard T H Chan School of Public Health, Boston, MA USA; 4 Department of Nutrition, Harvard T H Chan School of Public Health, Boston, MA USA; 5 Unit of Medical Epidemiology, Department of Surgical Sciences, Uppsala University, Uppsala, Sweden

**Keywords:** Risk factors, Genetic variation

## Abstract

We conducted a Mendelian randomization study to determine the potential causal associations of gallstone disease, diabetes, serum calcium, triglyceride levels, smoking and alcohol consumption with acute and chronic pancreatitis. Genetic variants associated with the exposures at *p* < 5 × 10^−8^ were selected from corresponding genome-wide association studies. Summary-level data for pancreatitis were obtained from the FinnGen consortium and UK Biobank. Univariable and multivariable Mendelian randomization analyses were performed and results from FinnGen and UK Biobank were combined using the fixed-effects meta-analysis method. Genetic predisposition to gallstone disease, type 2 diabetes and smoking initiation was associated with an increased risk of acute pancreatitis. The combined odds ratios (ORs) were 1.74 (95% confidence interval (CI), 1.57, 1.93) for gallstone disease, 1.14 (95% CI, 1.06, 1.21) for type 2 diabetes and 1.56 (95% CI, 1.32, 1.83) for smoking initiation. The association for type 2 diabetes attenuated after adjustment for gallstone disease. Genetic predisposition to gallstone disease and smoking initiation as well as higher genetically predicted serum calcium and triglyceride levels were associated with an increased risk of chronic pancreatitis. The combined ORs of chronic pancreatitis were 1.27 (95% CI, 1.08, 1.50) for gallstone disease, 1.86 (95% CI, 1.43, 2.43) for smoking initiation, 2.20 (95% CI, 1.30, 3.72) for calcium and 1.47 (95% CI, 1.23, 1.76) for triglycerides. This study provides evidence in support that gallstone disease, type 2 diabetes, smoking and elevated calcium and triglyceride levels are causally associated with the risk of acute or chronic pancreatitis.

## Introduction

Pancreatitis is a condition where pancreatic enzymes injure pancreatic tissue and lead to dysfunction of the gland as well as remote organs and systems. It is estimated that pancreatitis affected over 16 million people and caused nearly 4 million years lived with disability globally in 2017^1^. Acute pancreatitis is a common gastrointestinal cause of hospital admiss^2^. Approximately 8% of acute pancreatitis develop to chronic pancreatitis, which is associated with reduced quality of life and is linked to increased risk of metabolic disorders, such as diabetes and osteoporosis, as well as pancreatic cancer^3^. Identification of modifiable risk factors for pancreatitis is thus of great importance for pancreatitis prevention and lowering corresponding and consequent diseases and economic burdens.

Gallstone disease^2^, cigarette smoking^4,5^ and heavy alcohol drinking^4,5^ have been recognized as risk factors for both acute and chronic pancreatitis. However, about 20% of pancreatitis cases are idiopathic and with the etiological basis remaining undetermined^5^. Other possible risk factors for pancreatitis identified include hypercalcemia, mainly caused by hyperparathyroidism^6^, as well as hypertriglyceridemia^7,8^, type 2 diabetes^9^ and certain medications^5^. Most studies focused on the impact of these factors on acute pancreatitis. Whether above factors also play a role in chronic pancreatitis remains inconclusive. In addition, studies found that only heavy intake of alcohol (e.g. ≥5 drinks/day)^10^ or pathological excess of serum calcium^6^ increased the risk of pancreatitis. The effects of habitual moderate alcohol consumption (e.g. 1 drink/day) or high serum calcium levels within the normal range on pancreatitis are unclear. Available data on possible risk factors for pancreatitis are mostly based on observational studies that are prone to be challenged by residual confounding. Moreover, reverse causality could be of concern in such studies because levels of certain biomarkers and behaviors may be influenced by underlying disease. Therefore, it remains unclear whether previously reported risk factors are causally related to pancreatitis.

Using one or multiple genetic variants (e.g. single-nucleotide polymorphisms) as genetic instruments to mimic the genetically proxied effect of an exposure, the Mendelian randomization design can strengthen the causal inference of an exposure-outcome association in observational studies^11^. This approach has two major merits—reducing residual confounding and diminishing reverse causality, which can be achieved by the random allocation of genetic variants at conception and the fixed status of genetic variants that cannot modified by the onset or progression of the disease, respectively. Here, we conducted a two-sample Mendelian randomization study to determine the associations of genetically proxied gallstone disease, serum calcium levels, blood triglycerides levels, smoking initiation, alcohol consumption and type 2 diabetes with risk of acute and chronic pancreatitis.

## Results

### Acute pancreatitis

**Fig. 1:**
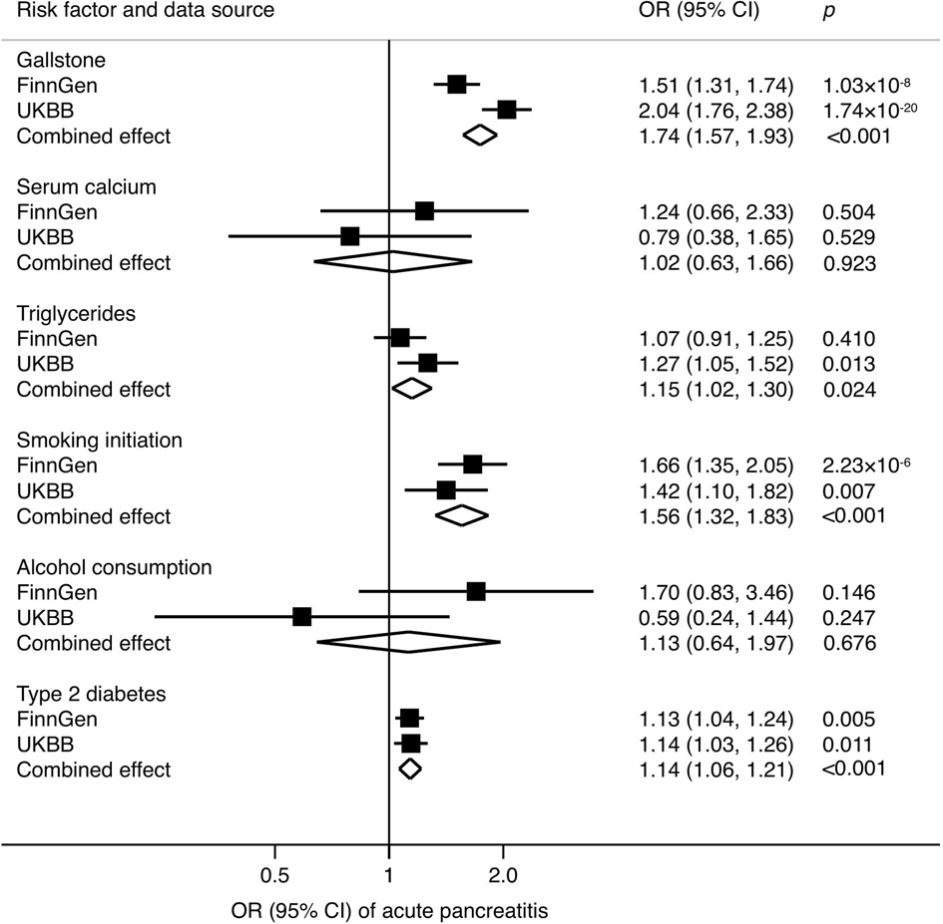
Combined effect^a^ of risk factors on acute pancreatitis in the FinnGen consortium and UK Biobank study. CI indicates confidence interval; OR odds ratio, UKBB UK Biobank. ^a^Estimates were based on the fixed-effect meta-analysis of odds ratio derived from the inverse-variance weighted method with random-effects.

Genetic predisposition to gallstone disease, smoking initiation and type 2 diabetes was associated with an increased risk of acute pancreatitis in both the FinnGen consortium and UK Biobank and the associations were statistically significant in the meta-analysis (Fig. 1). For one-unit or one SD increase in prevalence of these traits, the combined ORs and corresponding 95% CIs were 1.74 (95% CI, 1.57, 1.93) for gallstone disease, 1.56 (95% CI, 1.32, 1.83) for smoking initiation and 1.14 (95% CI, 1.06, 1.21) for type 2 diabetes. There was a suggestive positive association between genetically predicted triglyceride levels and acute pancreatitis in the combined analysis (OR_SD_, 1.15; 95% CI, 1.02, 1.30). There was limited data supporting associations of genetically proxied serum calcium levels and alcohol drinking with acute pancreatitis. Results remained consistent in sensitivity analyses (Supplementary Table 1). The moderate heterogeneity among SNPs was observed in the analysis of alcohol consumption in UK Biobank (*p* for Cochrane Q < 0.05) and pleiotropy was detected in the analysis of triglycerides in UK Biobank (*p* for the intercept in MR-Egger < 0.05).

**Fig. 2:**
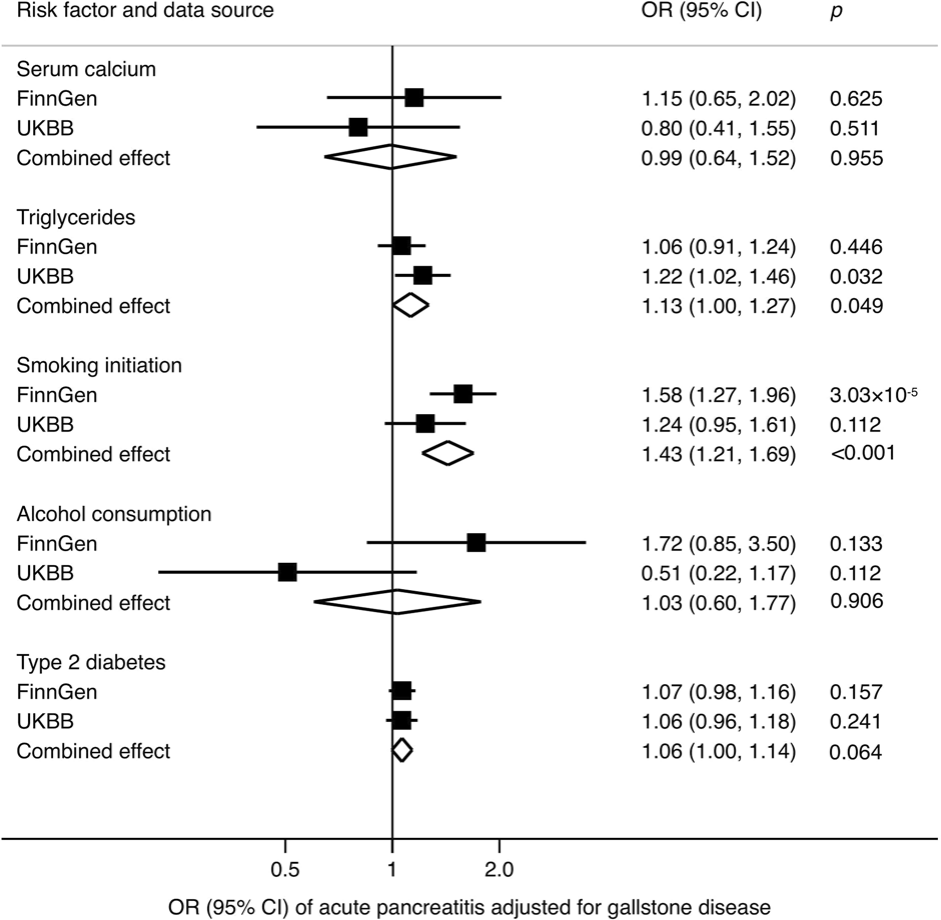
Combined effect^a^ of risk factors on acute pancreatitis after adjustment of gallstone disease in the FinnGen consortium and UK Biobank study. CI indicates confidence interval; OR odds ratio, UKBB UK Biobank. ^a^Estimates were based on the fixed-effect meta-analysis of odds ratio derived from the inverse-variance weighted method with random-effects adjusting for gallstone disease.

Associations adjusted for genetic liability to gallstone disease are displayed in Fig. 2. After adjustment for genetic liability to gallstone disease, the association between genetic predisposition to smoking initiation and acute pancreatitis persisted with an OR_SD_ of 1.43 (95% CI, 1.21, 1.69) in the combined analysis of FinnGen and UK Biobank. However, the association for genetic liability to type 2 diabetes attenuated largely and became nonsignificant. A suggestive association persisted for genetically predicted triglycerides (OR_SD_, 1.13; 95% CI, 1.00, 1.27).

### Chronic pancreatitis

**Fig. 3:**
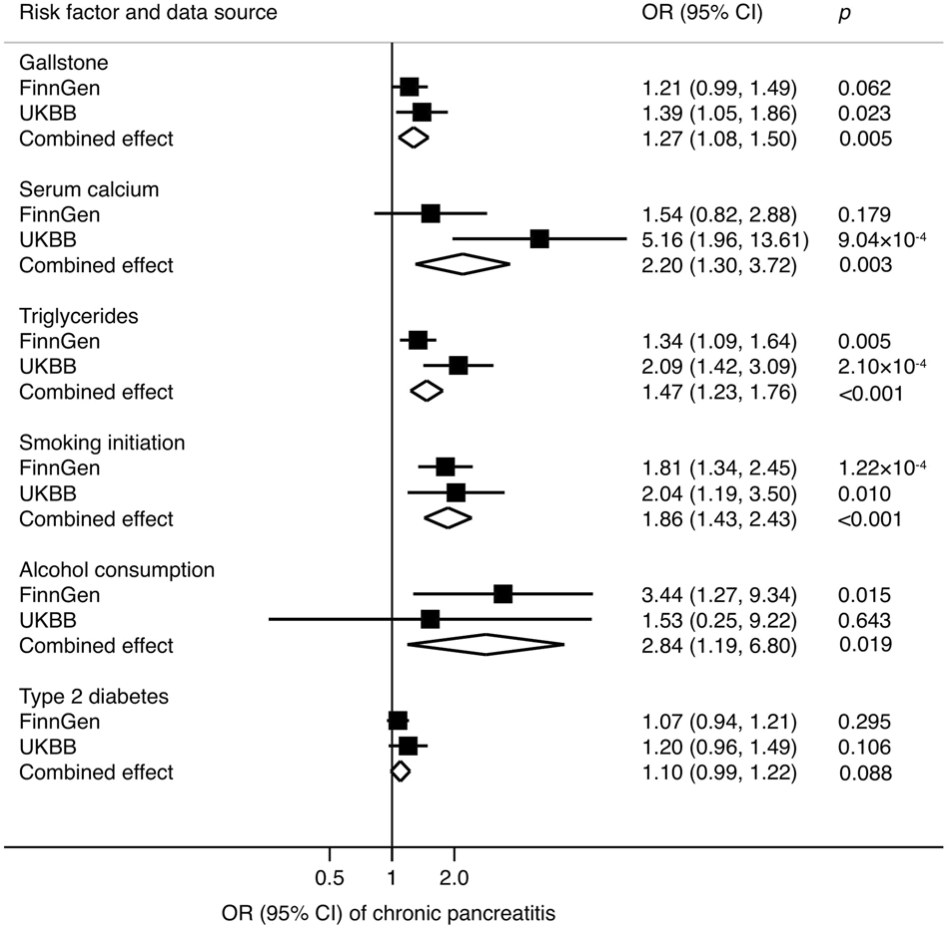
Combined effect^a^ of risk factors on chronic pancreatitis in the FinnGen consortium and UK Biobank study. CI indicates confidence interval; OR odds ratio, UKBB UK Biobank. ^a^Estimates were based on the fixed-effect meta-analysis of odds ratio derived from the inverse-variance weighted method with random-effects.

Genetic predisposition to gallstone disease, high serum calcium levels, high blood triglycerides and smoking initiation was associated with an increased risk of chronic pancreatitis (Fig. 3). These associations were consistent in both FinnGen consortium and UK Biobank. The combined ORs and corresponding 95% CIs of chronic pancreatitis were 1.27 (95% CI, 1.08, 1.50) for one-unit increase in prevalence of gallstone disease, 2.20 (95% CI, 1.30, 3.72) for one SD increase in serum calcium levels, 1.47 (95% CI, 1.23, 1.76) for one SD increase in blood triglycerides levels and 1.86 (95% CI, 1.43, 2.43) for one SD increase in prevalence of smoking initiation. There was a suggestive positive association between genetically predicted alcohol consumption and chronic pancreatitis in the meta-analysis of FinnGen consortium and UK Biobank (OR_SD_ 2.84; 95% CI, 1.19, 6.80). Genetic liability to type 2 diabetes was not associated with chronic pancreatitis. We observed significant heterogeneity among SNPs in the analysis for type 2 diabetes based on FinnGen data, but no possible pleiotropy (*p* for the intercept in MR-Egger > 0.05) (Supplementary Table 2).

**Fig. 4:**
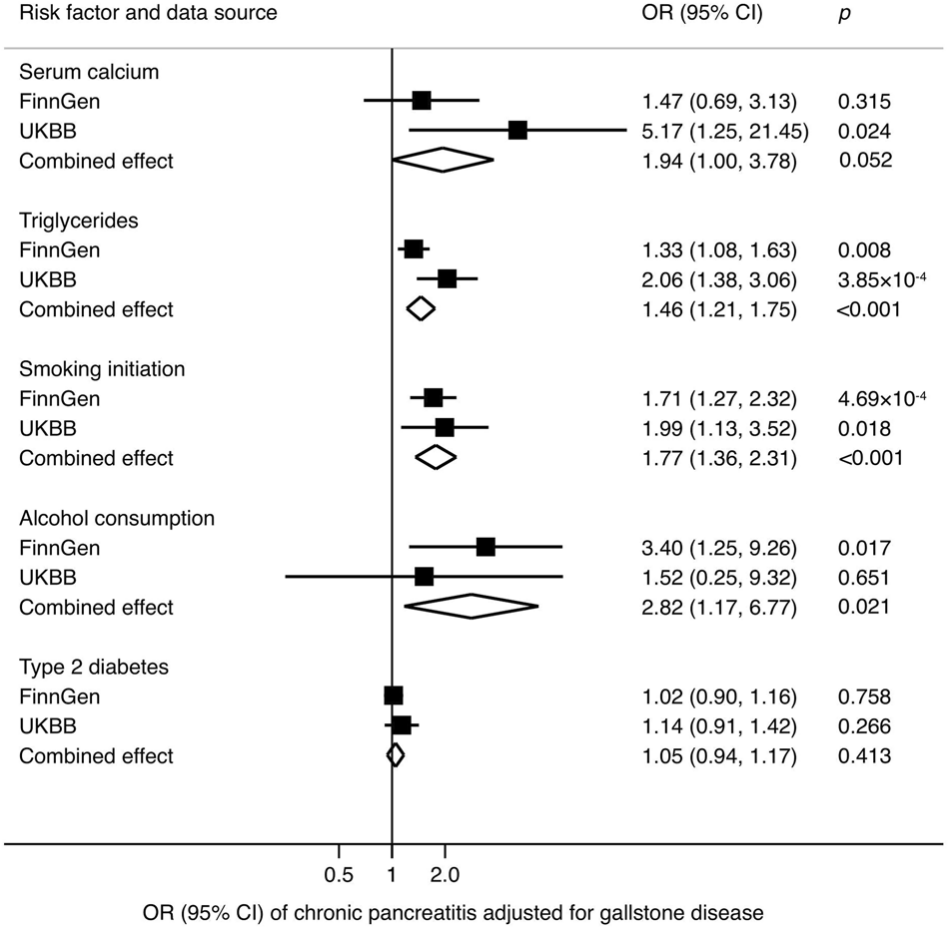
Combined effect^a^ of risk factors on chronic pancreatitis after adjustment of gallstone disease in the FinnGen consortium and UK Biobank study. CI indicates confidence interval; OR odds ratio, UKBB UK Biobank. ^a^Estimates were based on the fixed-effect meta-analysis of odds ratio derived from the inverse-variance weighted method with random-effects adjusting for gallstone disease.

Associations for genetic predisposition to elevated triglyceride levels, smoking initiation and alcohol consumption remained after adjustment for gallstone disease, albeit with slightly attenuated magnitudes. The OR_SD_s of chronic pancreatitis were 1.46 (95% CI, 1.21, 1.75) for triglycerides, 1.77 (95% CI, 1.36, 2.31) for smoking initiation and 2.82 (1.17, 6.77) for alcohol consumption (Fig. 4). The association between genetically predicted serum calcium and chronic pancreatitis became attenuated and borderline significant after adjustment for gallstone disease (OR_SD_ 1.94; 95% CI, 1.00, 3.76) (Fig. 4).

## Discussion

The present MR study found evidence that genetic predisposition to gallstone disease, type 2 diabetes and smoking initiation are associated with an increased risk of acute pancreatitis and that genetic predisposition to gallstone disease, high serum calcium levels, high blood triglyceride levels and smoking initiation are associated with an increased risk of chronic pancreatitis. The association for type 2 diabetes attenuated after adjusting for genetic liability to gallstone disease. There were suggestive positive associations of genetically predicted triglyceride levels with acute pancreatitis, and between genetic predisposition to moderate alcohol consumption and chronic pancreatitis.

Gallstone disease triggers pancreatitis, especially acute pancreatitis, which has been acknowledged in previous studies^2^. The present study strengthened the causality of this association based on human genetic data. Gallstone prevention and cholecystectomy can be even strongly recommended to lower the risk of acute pancreatitis among individuals with high risk of gallstone or those with gallstone disease. However, the role of gallstone disease in chronic pancreatitis remains controversial^2,12^. Some studies suggested that gallstones and biliary stricture or obstruction were complications but not causes of chronic pancreatitis^12^. The patients with symptomatic gallstones were usually treated by cholecystectomy and less likely to progress to chronic pancreatitis, whereas there was no recommended treatment for most patients with silent gallstones. It is estimated that ~4% of these patients develop symptoms including pancreatitis^13^, which might corroborate our finding suggesting that the formation of gallstone might increase the risk of chronic pancreatitis. This positive association was possibly supported by several underlying mechanisms, such as inflammation, obstruction of duct system, ampullary stenosis or sphincter of Oddi dysfunction, etc^12^.

Cigarette smoking has been established as a robust and independent risk factor for both acute and chronic pancreatitis^2,4,5^. The risk of pancreatitis increases in a dose-response manner in both men and women^14^. Our findings based on two independent populations further confirmed the causality of this association. A slight attenuation of the effect size after adjustment for gallstone disease suggests that gallstone disease may partly mediate the association between smoking and pancreatitis. The effect of smoking on pancreatitis has been suggested to be not only additive per se but also multiplicative when combined with alcohol^15^. Given a high correlation between smoking and alcohol drinking^10^, the detrimental effects of smoking on pancreatitis may be even larger than estimated. It is noted that the damage of smoking to pancreas seems to be reversible with the risk of pancreatitis decreasing^16^ and the progression of chronic pancreatitis slowing^15^ after adopting smoking cessation. Thus, reducing the prevalence of smoking initiation, lowering intensity of smoking and promoting smoking cessation can be strongly recommended as pancreatitis prevention strategies.

Alcohol abuse and heavy alcohol drinking (e.g. ≥5 drinks/day) pose a great danger on acute pancreatitis, recurrence of acute pancreatitis and chronic pancreatitis^5,10,15,17^. However, evidence on the association between habitual moderate alcohol consumption and pancreatitis is limited. A recent meta-analysis including 3618 pancreatitis cases out of 157,026 participants found a dose-response relationship between alcohol consumption and risk of acute and chronic pancreatitis in men and a non-linear association for acute pancreatitis in women^18^. In our study, there was limited data supporting an association between moderate alcohol consumption and acute pancreatitis. Nevertheless, we verified the possible causal association between habitual moderate alcohol consumption and chronic pancreatitis. The discrepancy between the present and previous study concerning acute pancreatitis might be caused by a very small portion (around 3%) of drinker with alcohol intake of over 1 drink (14 gram) per day in our study^19^ or a sex-difference in the association between alcohol drinking and acute pancreatitis noted in previous studies^18^.

Severe hypertriglyceridemia (>885 mg/dL; >10 mmol/L) with the prevalence of 1.7% among adults is a well-established cause of acute pancreatitis^20^. A recent study found that mild-to-moderate hypertriglyceridemia (>177 mg/dL; 2 mmol/L) was also associated with an elevated risk of acute pancreatitis^7^. In the North American Pancreatitis Study 2 continuation and validation study, around 13% of patients with chronic pancreatitis had hyperlipidemia^8^. Randomized controlled trials further proved the effectiveness of statin therapy in reducing risk of pancreatitis among patients with normal or mildly elevated triglyceride levels^21^. Findings of the present study supported such causal associations of moderately increased levels of triglycerides with ascended risk of pancreatitis. Notably, we noticed that the effect of hypertriglyceridemia appeared to be more profound on the development of chronic type compared to acute pancreatitis.

With regard to the association between serum calcium levels and pancreatitis, evidence is inconsistent with positive^6^ and null^22^ associations and based on patients with hyperparathyroidism. A population-based genetic study indicated that calcium sensing receptor polymorphisms showed relation to risk of chronic pancreatitis among the general population and individual with moderate to heavy alcohol consumption^23^. Our study with more pancreatitis cases revealed a possible association between physiologically-normally high serum calcium levels and chronic pancreatitis, which needs verification in future studies.

Type 2 diabetes has been associated with an increased risk of acute pancreatitis in large observational studies^9^, which was supported by our findings in the univariable MR model. However, the association did not persist after adjusting for gallstone disease, which implies that elevated risk of gallstone disease due to diabetes may primarily explain this positive association. Experimental evidence on antidiabetic medicine reported that a greater than 6-times increase in the risk of acute pancreatitis was found among individuals who use type 2 diabetes medications, such as sitagliptin (a dipeptidyl peptidase 4 inhibitor) and exenatide (a glucagon-like peptide 1 analogue)^24^. Thus, whether type 2 diabetes play an independent role in acute pancreatitis needs more investigations.

The major strength of the present study is the MR design which can minimize residual confounding and diminish reverse causality. Another strength is the consistent results in two independent populations, which supported the robustness and reliability of the associations of several modifiable risk factors and with acute and chronic pancreatitis. A limitation of our study is possible pleiotropy. However, our results remained quite stable across all MR methods with different assumptions and there was no evidence of pleiotropy, except in the analysis of triglycerides based on UK Biobank data. Population stratification bias is less likely to affect our results since the used genome-wide association studies were based on individuals of solely European origin, except for the genome-wide association study on type 2 diabetes where around 80% recruited participants were of European descent. In addition, adjustment was made for population structure through genetic principal components in these genome-wide association studies. However, this population restriction might confine the generalizability of our findings to other populations.

In summary, the present MR study suggested that genetic predisposition to gallstone disease and smoking initiation might be causally associated with an increased risk of acute pancreatitis and that genetic predisposition to gallstone disease, high calcium and triglycerides levels and smoking initiation might be causally associated with an increased risk of chronic pancreatitis. Type 2 diabetes might influence acute pancreatitis mostly via gallstone disease. The suggestive associations between triglycerides and acute pancreatitis, and between alcohol consumption and chronic pancreatitis need verification.

## Methods

### Study design

**Fig. 5:**
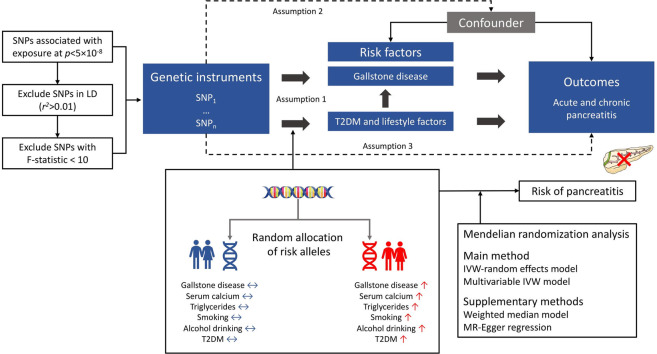
Study design overview and assumptions of the Mendelian randomization framework. IVW inverse-variance weighted, LD linkage disequilibrium, SNPs single-nucleotide polymorphisms, T2DM type 2 diabetes mellitus. Assumption 1 indicates that the genetic variants proposed as instrumental variables should be robustly associated with the risk factor of interest; assumption 2 indicates that the used genetic variants should not be associated with potential confounders, and assumption 3 indicates that the selected genetic variants should affect the risk of the outcome merely through the risk factor, not via alternative pathways.

Study design overview is shown in Fig. 5, and detailed information on used studies and genetic consortia in Table 1. All studies had been approved by a relevant ethical review board and participants had given informed consent. The present MR study has been approved by the Swedish Ethical Review Authority.

**Table 1:** Used genome-wide association studies and consortia in the present study.

Trait	Unit	SNPs	F-statistic	Participants	Adjustments	PubMed ID or web-link
Serum calcium	mg/dL (rescaled to 1 SD, equivalent to 0.5 mg/dL)	7	74	Up to 61,079 European-descent individuals	Age, sex and study-specific covariates (e.g., principal components and study center)	24068962
Blood triglycerides	SD	440	117	441,016 European-descent individuals	Age, sex and a binary variable denoting the genotyping chip	32203549
Smoking initiation	SD in prevalence of smoking initiation	378	46	1,232,091 European-descent individuals	Age, sex and the first ten genetic principal components	30643251
Alcohol consumption	SD increase of log-transformed alcoholic drinks/week	99	65	941,280 European-descent individuals	Age, sex and the first ten genetic principal components	30643251
Type 2 diabetes	One-unit in prevalence of type 2 diabetes	558	91	228,499 type 2 diabetes cases and 1,178,783 non-cases of multiancestries	Age, sex and the first ten genetic principal components	32541925
Gallstone disease	One-unit in prevalence of gallstone disease	32	130	27,174 gallstone disease cases and 736,838 non-cases of European ancestry	Unknown	30504769
Acute pancreatitis	—	—	—	1292 cases and 359,902 non-cases of British ancestry	Age, sex and up to 20 genetic principal components	UK Biobank (http://www.nealelab.is/uk-biobank)
Chronic pancreatitis	—	—	—	246 cases and 360,948 non-cases of British ancestry	Age, sex and up to 20 genetic principal components	UK Biobank (http://www.nealelab.is/uk-biobank)
Acute pancreatitis	—	—	—	1762 cases and 121,348 non-cases of Finnish ancestry	Age, sex, 10 genetic principal components and genotyping batch	FinnGen consortium (https://www.finngen.fi/fi)
Chronic pancreatitis	—	—	—	914 cases and 121,348 non-cases of Finnish ancestry	Age, sex, 10 genetic principal components and genotyping batch	FinnGen consortium (https://www.finngen.fi/fi)

PubMed ID indicates PubMed identifier.

*SD* standard deviation, *SNP* single-nucleotide polymorphisms.

### Genetic instrument selection

Genetic instruments for gallstone disease^25^, serum calcium^26^, blood triglycerides^27^, cigarette smoking^19^, alcohol drinking^19^ and type 2 diabetes^28^ were selected at genome-wide significance threshold (*p* < 5 × 10^−8^) from corresponding genome-wide association studies. Independent SNPs for each trait were defined by r^2^ > 0.01 and clump window <10 kb and correlated SNPs (e.g. linkage disequilibrium) with the lowest *p*-value was retained. Linkage disequilibrium among SNPs for each risk factor was calculated based on 1000 genomes LD reference panel (European population) using ﻿the PLINK clumping approach. The Cragg-Donald F-statistic was estimated for each SNP and all F-statistics were over 10.

### Outcome data source

Summary-level data for acute and chronic pancreatitis were obtained from the FinnGen consortium^29^ and UK Biobank study^30^. We used the R3 release of the FinnGen consortium data with 1762 acute pancreatitis (defined by K85 in ICD-10 and 5770 in ICD-8) cases, 914 chronic pancreatitis (defined by K861/K860 in ICD-10 and 5771 in ICD-8) cases and nearly 122,000 non-cases of Finnish ancestry. In this dataset, certain individuals were excluded, such as those with ambiguous gender, high genotype missingness (>5%), excess heterozygosity (±4 standard deviation) and non-Finnish ancestry. In quality control, genetic variants with high missingness (>2%), low Hardy-Weinberg equilibrium *p*-value (*p* < 5 × 10^−6^) and minor allele count, minor allele counts <3 were excluded. Association tests had been adjusted for age, sex, 10 genetic principal components and genotyping batch. Data from UK Biobank were based on the second wave of analyses of UK Biobank from Neale lab including 361,194 individuals after excluding those of non-European ancestry, closely related individuals (or at least one of a related pair of individuals), individuals with sex chromosome aneuploidies and individuals who had withdrawn consent. In total, 1292 acute and 246 chronic pancreatitis was defined by the ICD-10 code K85 and K861/K860, respectively. In quality control stage, Neale lab excluded SNPs with minor allele frequency <0.1% and Hardy-Weinberg *p*-value < 1 × 10^−10^, an INFO score <0.8. Association tests had been adjusted for age, sex and up to 20 principal components.

### Statistical analysis

The univariable and multivariable random-effects inverse-variance weighted model was used as the main analysis. Derived estimates from the FinnGen consortium and UK Biobank study were combined using the fixed-effects meta-analysis method. To assess whether gallstone disease is a mediator in the pathway from lifestyle factors to pancreatitis, multivariable MR analyses adjusting for gallstone disease status were performed to examine the gallstone-free association of serum calcium, blood triglycerides, smoking, alcohol drinking and type 2 diabetes with pancreatitis. The weighted median approach and MR-Egger regression was used as sensitivity analyses. The weighted median model provides consistent estimates at the prerequisite that ≥50% of the weight in the analysis comes from valid instrumental variables^31^. The MR-Egger regression can detect and correct for directional pleiotropy; however, it compromises power^32^. The *p-*value for the MR-Egger intercept was used to indicate directional pleiotropy and the *p*-value < 0.05 indicated possible pleiotropy. We used Cochrane’s Q value to estimate the heterogeneity among SNPs. Odds ratios (ORs) and corresponding confidence intervals (CIs) of pancreatitis were scaled to one-unit increase shown in Table 1. For serum calcium, we rescaled the estimate expressed in mg/dL to a one standard deviation (SD), equivalent to 0.5 mg/dL. The Bonferroni method was used to correct for multiple testing in univariable MR models. The combined association with two-sided *p*-values < 0.008 (where α = 0.05/6) were deemed statistically significant. Associations with *p*-values between 0.05 and 0.008 were regarded as suggestive associations, requiring confirmation. All analyses were performed using the TwoSampleMR^33^ and Mendelian randomization^34^ packages in R Software 3.6.0 (R Core Team. R Foundation for Statistical Computing. Vienna, Austria. 2019. https://www.R-project.org).

### Reporting summary

Further information on experimental design is available in the Nature Research Reporting Summary linked to this paper.

## Supplementary information

Supplementary Information

Reporting Summary

## Data Availability

All used data have been uploaded to the OSF data respiratory (available at: https://osf.io/cznqx/)
