# Supplementary material for: Gallstone disease, diabetes, calcium, triglycerides, smoking and alcohol consumption and pancreatitis risk: Mendelian randomization study
Source: NPJ Genom Med. 2021 Mar 29;6:27. doi: 10.1038/s41525-021-00189-6 (PMC8007637; doi:10.1038/s41525-021-00189-6)
Supplement: Supplementary file 1 — Supplementary Information [file 41525_2021_189_MOESM1_ESM.pdf]

Supporting information

Modifiable pathways for pancreatitis: a Mendelian randomization investigation

Shuai Yuan, Edward L Giovannucci, Susanna C. Larsson

Supplementary table 1. Associations of risk factors with acute pancreatitis in sensitivity analyses

Supplementary table 2. Associations of risk factors with chronic pancreatitis in sensitivity analyses

Supplementary table 1. Associations of risk factors with acute pancreatitis in sensitivity analyses

Risk factor	Used SNPs	Cochrane Q	P_Q	Weight median method			MR-Egger regression			
				OR	95% CI	*P*	OR	95% CI	*P*	$P_{intercept}$
**FinnGen**										
Gallstone disease	30	36.77	0.152	1.60	1.35, 1.91	1.2E-7	1.64	1.31, 2.06	2.0E-4	0.370
Serum calcium	7	8.17	0.226	1.56	0.83, 2.90	0.164	1.77	0.52, 6.03	0.404	0.530
Triglycerides	400	441.04	0.072	0.98	0.75, 1.28	0.879	0.93	0.73, 1.17	0.525	0.112
Smoking initiation	352	339.36	0.662	1.95	1.42, 2.67	3.1E-5	2.79	1.14, 6.87	0.026	0.244
Alcohol consumption	91	90.73	0.458	2.05	0.68, 6.16	0.203	2.71	0.46, 15.9	0.272	0.571
Type 2 diabetes	528	567.76	0.107	1.09	0.93, 1.29	0.288	1.18	0.99, 1.41	0.072	0.622
**UKBB**										
Gallstone disease	30	29.19	0.455	1.81	1.45, 2.26	1.9E-7	1.89	1.47, 2.43	3.5E-5	0.451
Serum calcium	7	8.00	0.238	1.08	0.51, 2.29	0.849	2.17	0.67, 7.04	0.255	0.102
Triglycerides	431	461.26	0.136	1.02	0.74, 1.39	0.918	0.80	0.60, 1.06	0.120	4.3E-5
Smoking initiation	366	393.09	0.149	1.31	0.90, 1.92	0.158	0.68	0.24, 1.96	0.479	0.163
Alcohol consumption	96	141.30	0.001	0.36	0.10, 1.21	0.098	0.27	0.05, 1.60	0.153	0.324
Type 2 diabetes	552	608.52	0.042	1.15	0.95, 1.41	0.158	1.01	0.82, 1.24	0.936	0.186

CI indicates confidence interval; OR, odds ratio; SNPs, single nucleotide polymorphisms; UKBB, UK Biobank.

Supplementary table 2. Associations of risk factors with chronic pancreatitis in sensitivity analyses

Risk factor	Used SNPs	Cochrane Q	P_Q	Weighted median method			MR-Egger regression			
				OR	95% CI	*P*	OR	95% CI	*P*	$P_{intercept}$
**FinnGen**										
Gallstone disease	30	41.36	0.064	1.37	1.07, 1.74	0.011	1.42	1.03, 1.96	0.042	0.229
Serum calcium	7	4.26	0.642	1.40	0.59, 3.31	0.445	1.46	0.37, 5.75	0.616	0.930
Triglycerides	400	376.58	0.773	1.28	0.87, 1.88	0.216	1.14	0.83, 1.56	0.413	0.181
Smoking initiation	352	372.85	0.202	1.72	1.10, 2.69	0.018	2.97	0.83, 10.67	0.096	0.435
Alcohol consumption	91	94.23	0.359	4.30	0.97, 19.12	0.056	4.37	0.36, 52.45	0.248	0.837
Type 2 diabetes	528	589.22	0.031	1.19	0.97, 1.47	0.093	1.19	0.93, 1.52	0.178	0.338
**UKBB**										
Gallstone disease	30	21.39	0.845	1.41	0.87, 2.30	0.167	1.12	0.64, 1.96	0.685	0.351
Serum calcium	7	2.79	0.835	8.18	1.50, 44.55	0.015	18.90	1.37, 260.39	0.079	0.301
Triglycerides	431	414.90	0.679	1.07	0.54, 2.14	0.847	1.35	0.72, 2.51	0.347	0.072
Smoking initiation	366	363.12	0.518	1.61	0.75, 3.48	0.225	0.24	0.03, 2.28	0.215	0.056
Alcohol consumption	96	114.92	0.080	4.23	0.19, 95.28	0.364	4.77	0.14, 166.76	0.391	0.468
Type 2 diabetes	552	558.31	0.394	1.19	0.81, 1.75	0.386	0.93	0.59, 1.45	0.746	0.205

CI indicates confidence interval; OR, odds ratio; SNPs, single nucleotide polymorphisms; UKBB, UK Biobank.
