# Supplementary material for: Gallstone disease, diabetes, calcium, triglycerides, smoking and alcohol consumption and pancreatitis risk: Mendelian randomization study
Source: NPJ Genom Med. 2021 Mar 29;6:27. doi: 10.1038/s41525-021-00189-6 (PMC8007637; doi:10.1038/s41525-021-00189-6)
Supplement: Supplementary file 2 — Reporting Summary [file 41525_2021_189_MOESM2_ESM.pdf]

nature research

Corresponding author(s): Susanna C. Larsson
Last updated by author(s): Feb 11, 2021

Reporting Summary

Nature Research wishes to improve the reproducibility of the work that we publish. This form provides structure for consistency and transparency in reporting. For further information on Nature Research policies, see our Editorial Policies and the Editorial Policy Checklist.

Statistics

For all statistical analyses, confirm that the following items are present in the figure legend, table legend, main text, or Methods section.

n/a	Confirmed	
☐	☒	The exact sample size (*n*) for each experimental group/condition, given as a discrete number and unit of measurement
☐	☒	A statement on whether measurements were taken from distinct samples or whether the same sample was measured repeatedly
☐	☒	The statistical test(s) used AND whether they are one- or two-sided<br>*Only common tests should be described solely by name; describe more complex techniques in the Methods section.*
☐	☒	A description of all covariates tested
☐	☒	A description of any assumptions or corrections, such as tests of normality and adjustment for multiple comparisons
☐	☒	A full description of the statistical parameters including central tendency (e.g. means) or other basic estimates (e.g. regression coefficient) AND variation (e.g. standard deviation) or associated estimates of uncertainty (e.g. confidence intervals)
☐	☒	For null hypothesis testing, the test statistic (e.g. *F*, *t*, *r*) with confidence intervals, effect sizes, degrees of freedom and *P* value noted<br>*Give P values as exact values whenever suitable.*
☒	☐	For Bayesian analysis, information on the choice of priors and Markov chain Monte Carlo settings
☒	☐	For hierarchical and complex designs, identification of the appropriate level for tests and full reporting of outcomes
☒	☐	Estimates of effect sizes (e.g. Cohen's *d*, Pearson's *r*), indicating how they were calculated

Our web collection on statistics for biologists contains articles on many of the points above.

Software and code

Policy information about availability of computer code

Data collection	NA
Data analysis	We used the TwoSampleMR and Mendelianrandomization packages in R Software 3.6.0 (R Core Team. R Foundation for Statistical Computing. Vienna, Austria. 2019. https://www.R-project.org) to analyze the data.

For manuscripts utilizing custom algorithms or software that are central to the research but not yet described in published literature, software must be made available to editors and reviewers. We strongly encourage code deposition in a community repository (e.g. GitHub). See the Nature Research guidelines for submitting code & software for further information.

Data

Policy information about availability of data

All manuscripts must include a data availability statement. This statement should provide the following information, where applicable:

- Accession codes, unique identifiers, or web links for publicly available datasets
- A list of figures that have associated raw data
- A description of any restrictions on data availability

The datasets analyzed in this study are publicly available summary statistics. Summary level data for studied exposures can be obtained through cited references. The GWAS data for pancreatitis are available online: the UK Biobank study (http://www.nealelab.is/uk-biobank) and the FinnGen consortium (https://www.finngen.fi/fi).

Field-specific reporting

Please select the one below that is the best fit for your research. If you are not sure, read the appropriate sections before making your selection.

☒ Life sciences ☐ Behavioural & social sciences ☐ Ecological, evolutionary & environmental sciences

For a reference copy of the document with all sections, see nature.com/documents/nr-reporting-summary-flat.pdf

Life sciences study design

All studies must disclose on these points even when the disclosure is negative.

Sample size	No sample size calculation was performed prior this study. We used data from all included individuals.
Data exclusions	Only independent SNPs were used. For outcomes, certain individuals were excluded, such as those with ambiguous gender, high genotype missingness (>5%), excess heterozygosity (±4 standard deviation) and non-Finnish ancestry in the FinnGen consortium. The second wave of analyses of UK Biobank from Neale lab including 361 194 individuals after excluding those of non-European ancestry, closely related individuals (or at least one of a related pair of individuals), individuals with sex chromosome aneuploidies, and individuals who had withdrawn consent.
Replication	Results were replicated in two independent datasets.
Randomization	Randomization is not applicable to our study.
Blinding	Blinding is not applicable to our study.

Reporting for specific materials, systems and methods

We require information from authors about some types of materials, experimental systems and methods used in many studies. Here, indicate whether each material, system or method listed is relevant to your study. If you are not sure if a list item applies to your research, read the appropriate section before selecting a response.

Materials & experimental systems

n/a	Involved in the study	
☒	☐	Antibodies
☒	☐	Eukaryotic cell lines
☒	☐	Palaeontology and archaeology
☒	☐	Animals and other organisms
☐	☒	Human research participants
☒	☐	Clinical data
☒	☐	Dual use research of concern

Methods

n/a	Involved in the study	
☒	☐	ChIP-seq
☒	☐	Flow cytometry
☒	☐	MRI-based neuroimaging

Human research participants

Policy information about studies involving human research participants

Population characteristics	We used the R3 release of the FinnGen consortium data with 1762 acute pancreatitis (defined by K85 in ICD-10 and 5770 in ICD-8) cases, 914 chronic pancreatitis (defined by K861/K860 in ICD-10 and 5771 in ICD-8) cases and nearly 122 000 non-cases of Finnish ancestry. In the UK Biobank, In total, 1292 acute and 246 chronic pancreatitis was defined by the ICD-10 code K85 and K861/K860, respectively, and the controls were over 360000 individuals.
Recruitment	Individuals who pass inclusion requirements were recruited in the UK Biobank and FinnGen consortium.
Ethics oversight	As all analyses were based on publicly available summary statistics, no ethical approval was required.

Note that full information on the approval of the study protocol must also be provided in the manuscript.
